# Supplementary material for: Digital spatial profiling of intraductal papillary mucinous neoplasms: Toward a molecular framework for risk stratification
Source: Sci Adv. 2023 Mar 17;9(11):eade4582. doi: 10.1126/sciadv.ade4582 (PMC10022906; doi:10.1126/sciadv.ade4582)
Supplement: Supplementary file 1 — Fig S1 to S8 Legend for data file S1 [file sciadv.ade4582_sm.pdf]

Science Advances
AAAS

Supplementary Materials for

Digital spatial profiling of intraductal papillary mucinous neoplasms: Toward a molecular framework for risk stratification

Matthew K. Iyer *et al.*

Corresponding author: Peter J. Allen, peter.allen@duke.edu

Sci. Adv. **9**, eade4582 (2023)
DOI: 10.1126/sciadv.ade4582

The PDF file includes:

Figs. S1 to S8
Legend for data file S1

Other Supplementary Material for this manuscript includes the following:

Data file S1

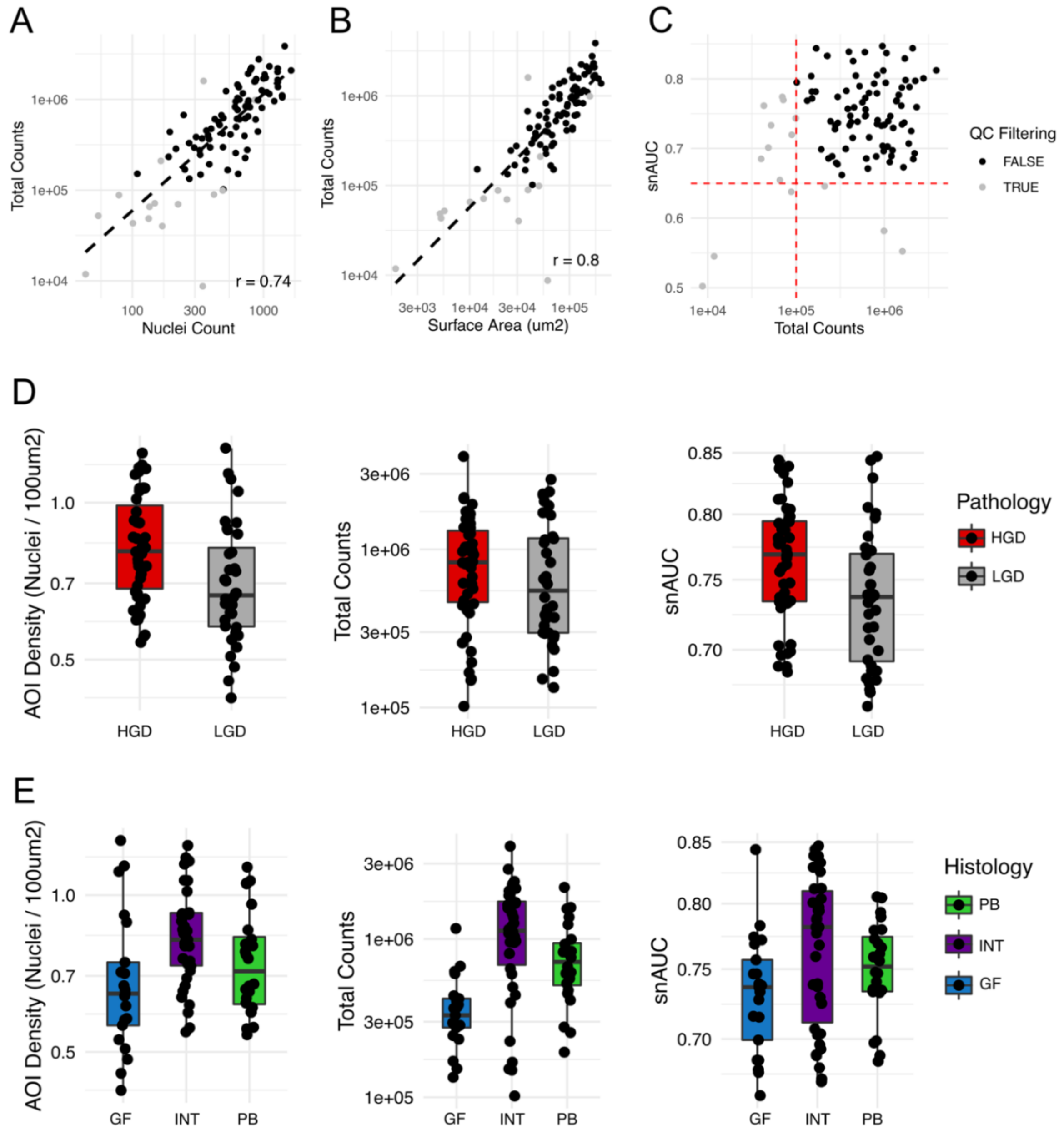

Fig. S1: QC analysis of epithelial AOIs. (**A-B**) Scatter plots of AOI QC parameters showing filtered AOIs (grey) and retained AOIs (black) based on nuclei count (**A**) and surface area (**B**) relative to total deduplicated sequencing counts. (**C**) Scatter plot depicting total deduplicated sequencing counts versus snAUC with dotted red lines indicating filtering cutoffs, and filtered and retailed AOIs indicated by grey and black dots, respectively. (**D**) Box plots by grade of dysplasia (red = HGD, grey = LGD) comparing the distribution of (*left*) AOI density (Nuclei / Surface Area), (*middle*) total deduplicated sequencing counts, and (*right*) snAUC. (**E**) Box plots by epithelial subtype (blue = GF, purple = INT, green = PB) comparing the distribution of (*left*) AOI density (Nuclei / Surface Area), (*middle*) total deduplicated sequencing counts, and (*right*) snAUC.

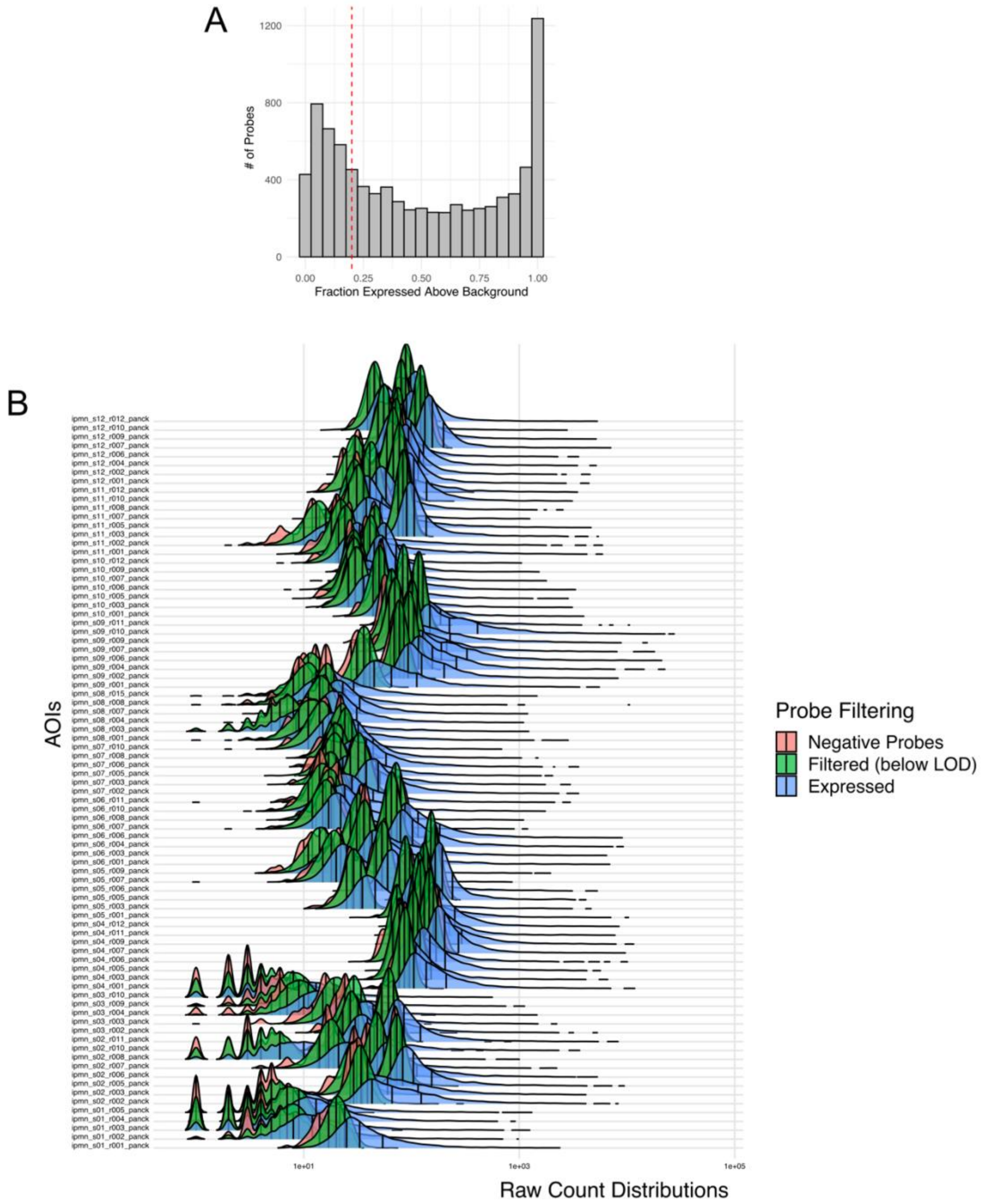

Fig. S2: Probe QC analysis. (**A**) Histogram depicting the fraction of probes with counts above the limit of detection across all AOIs. Dotted red line indicates filtering cutoff. (**B**) Ridge plot (layered density plot) showing raw (unnormalized) count distributions for all AOIs. Probes groups include background (red), filtered (green), and expressed (blue).

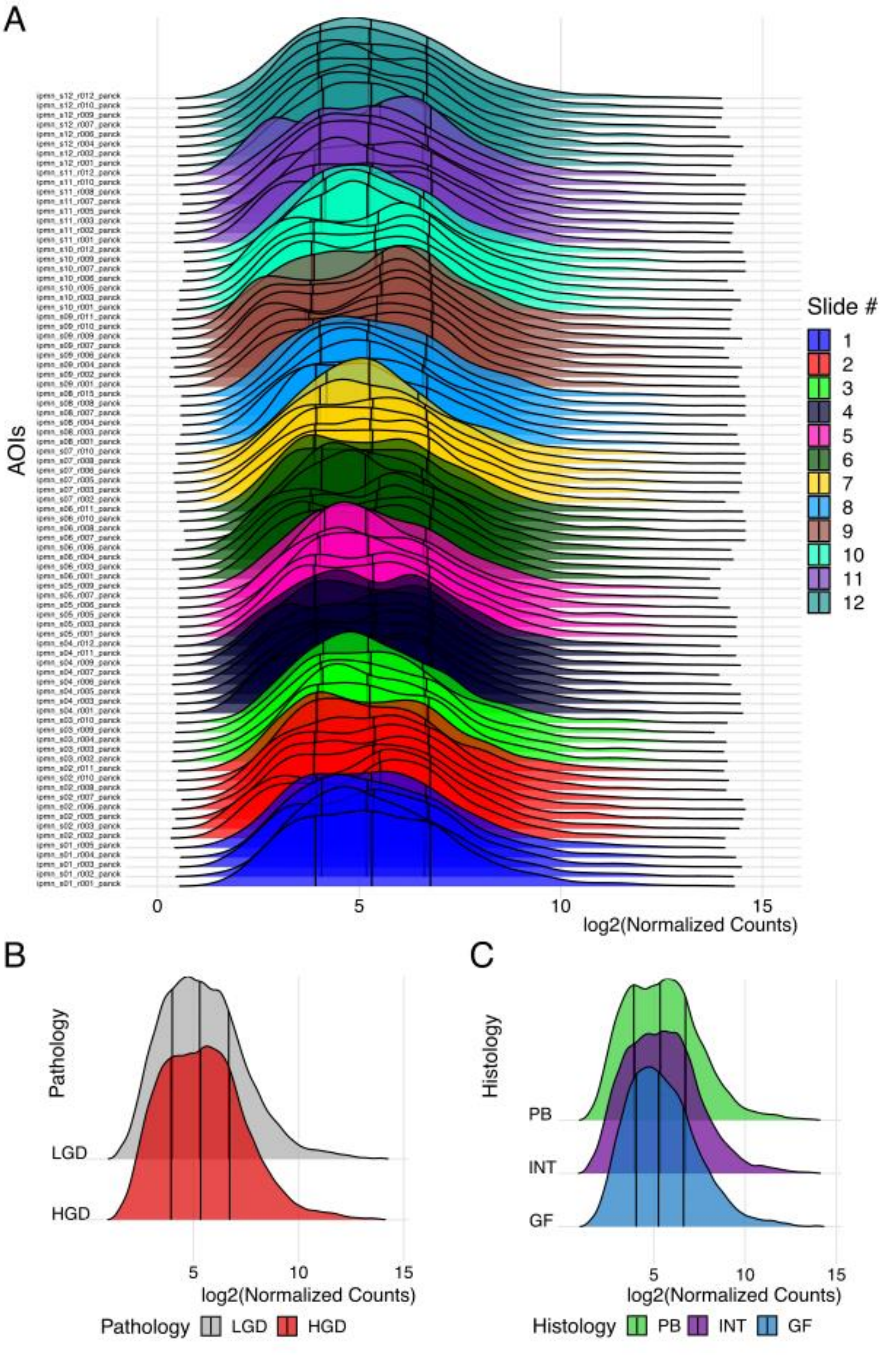

Fig. S3: Ridge plots of filtered log-normalized count distributions (x axis), (**A**) Individual AOIs colored by slide identifier. (**B**) Aggregated AOIs dichotomized by LGD or HGD. (**C**) Aggregated AOIs separated by PB, INT, and GF subtypes.

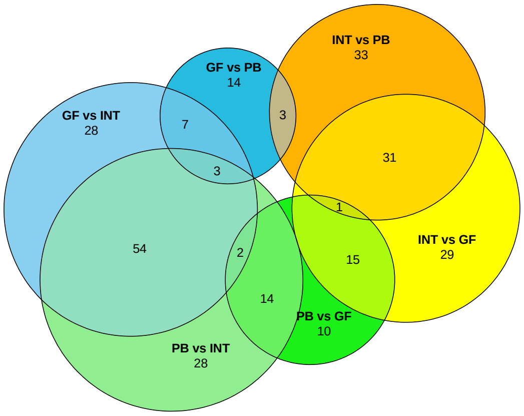

Fig. S4: Area-proportional Euler diagram showing the intersection of DE gene sets from pairwise comparisons of PB, INT, and GF AOIs. Plot generated using the *eulerr* package by J. Larsson (https://CRAN.R-project.org/package=eulerr).

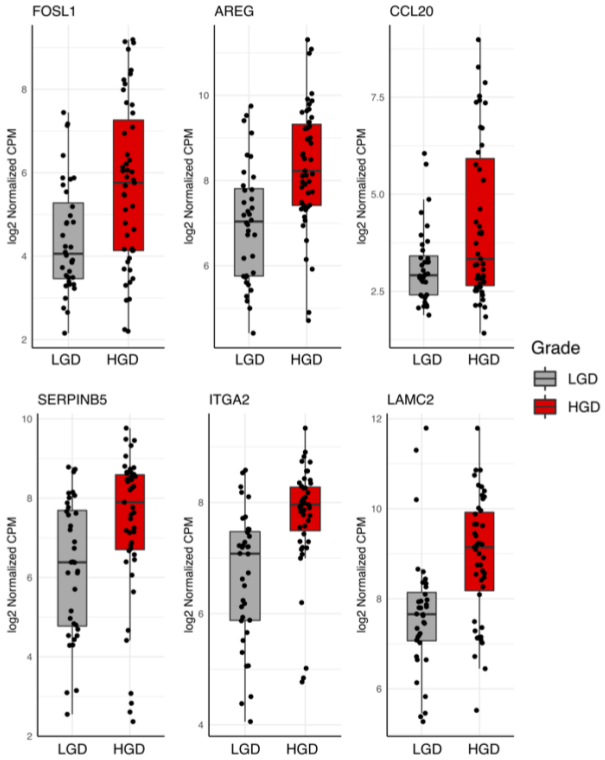

Fig. S5: Boxplots of differentially expressed genes in HGD versus LGD IPMN that were also represented within the CPTAC PDAC RNA-Seq dataset and associated with unfavorable prognosis in PDAC (Human Protein Atlas).

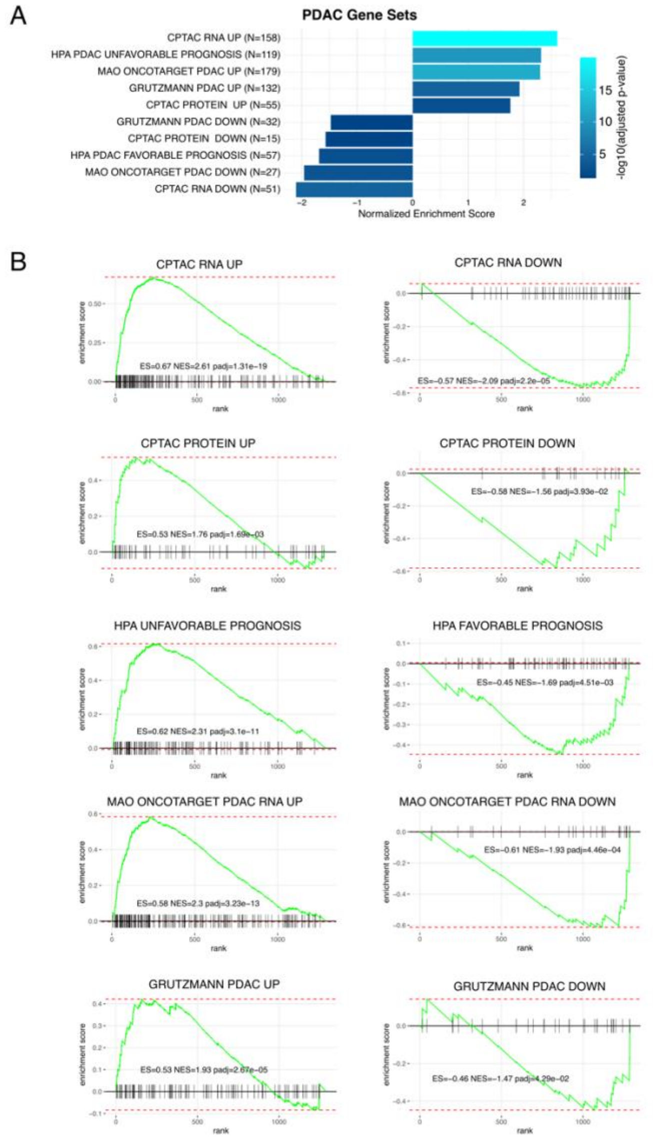

Fig. S6: GSEA analysis of AOIs with HGD versus LGD based on curated external gene sets. **(A)** Summary bar plot showing normalized enrichment score of each gene set. Bar colors reflect adjusted p-value. All adjusted p-values were <0.05. **(B)** GSEA plots of each individual analysis depicted in **(A)**.

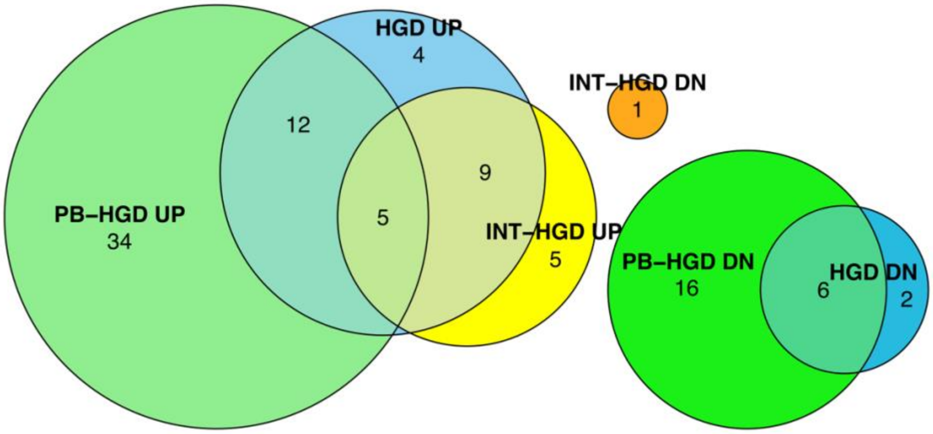

Fig. S7: Area-proportional Euler diagram showing the intersection of DE gene sets from multiple comparisons: HGD versus LGD, PB-HGD versus PB-LGD, and INT-HGD versus INT-LGD. Plot generated using the *eulerr* package by J. Larsson (https://CRAN.R-project.org/package=eulerr).

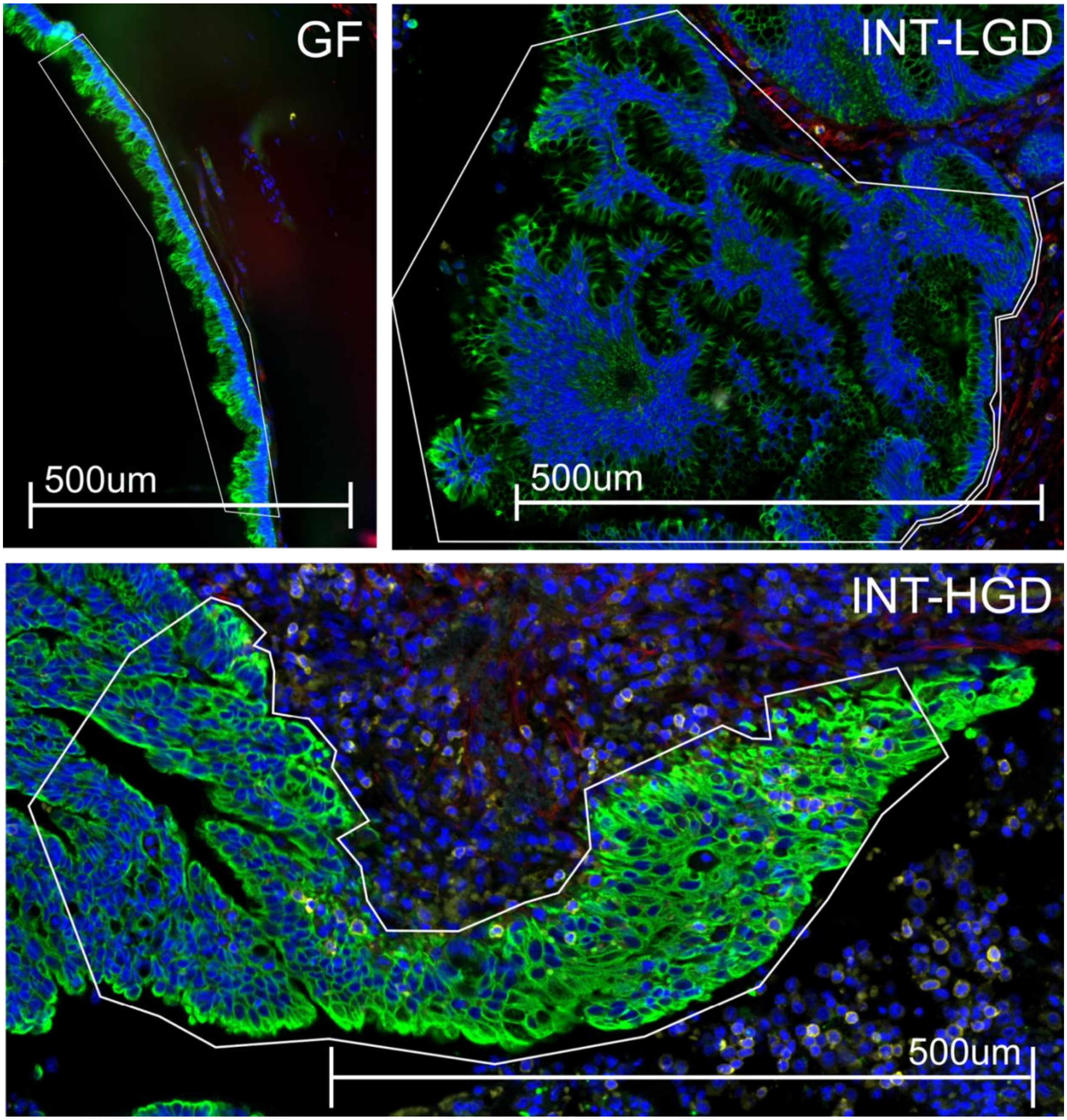

Fig. S8: Example AOIs from slide 2 harboring GF, INT-LGD, and INT-HGD subtypes.

Supplementary Tables

Table	Name	Description
Table S1	Unfiltered Metadata	Metadata associated with each AOI
Table S2	Probe Counts	Unfiltered raw probe counts and associated probe metadata
Table S3	Filtered Metadata	Metadata associated with AOIs that pass QC filters
Table S4	Gene Metadata	Filtered gene metadata associated with downstream analysis
Table S5	Gene Expression	Background subtracted quantile normalized log2 transformed gene counts
Table S6	DE Analysis	Compilation of all differential expression analyses performed
Table S7	GSEA PDAC	GSEA across curated pancreatic ductal adenocarcinoma gene sets
Table S8	GSEA Hallmark	GSEA across MSigDB Hallmark gene sets
Table S9	Coexpression Network Nodes	Nodes of coexpression network with associated properties
Table S10	Coexpression Network Edges	Edges between nodes of coexpression network with associated properties
Table S11	Community Enrichments	Enrichment of network communities with MSigDB Hallmark, GO:BP, and PDAC gene sets
**Tables S1 to S11 can be found in Data File S1**		
